# Age-associated outcomes in cardiogenic shock supported with microaxial flow pumps

**DOI:** 10.1016/j.aicoj.2026.100104

**Published:** 2026-06-24

**Authors:** Aurore Ughetto, Clement Delmas, Monika Fürholz, Laurent Bonello, Guillaume Leurent, Wiktor Kuliczkowski, Ana Hurtado, Philippe Gaudard, Chloé Tassain, François Xavier Herion, Agnieszka tycinska, Benedikt Schrage, Marta Zaleska Kociecka, Frederic Bouisset, Matias Jacomet, Mikolaj Blaziak, Aneta Klotzka, Antoine Beurton, Miloud Cherbi, for the IMPACT Investigators

**Affiliations:** a Department of Cardiothoracic Anaesthesia and Critical Care Medicine, Montpellier University Hospital, Montpellier, France; b INSERM, CNRS, Université de Montpellier, PHYMEDEXP, Montpellier, France; c Intensive Cardiac Care Unit, Cardiology Department, Rangueil University Hospital, Toulouse, France; d Recherche et Enseignement en Insuffisance Cardiaque Avancée, Assistance et Transplantation (REICATRA), Institut Saint Jacques, CHU de Toulouse, Toulouse, France; e Department of Cardiology, Inselspital, Bern, Switzerland; f Assistance Publique-Hôpitaux de Marseille, Department of Cardiology, Hôpital Nord, Marseille, France; g Department of Cardiology, University of Rennes, CHU Rennes, Inserm, LTSI - UMR 1099, Rennes, France; h Institute of Heart Diseases, Faculty of Medicine, Wroclaw Medical University, Wrocław, Poland, University Hospital in Wroclaw, Wrocław, Poland; i Department of Critical Care, Harefield Hospital, Royal Brompton and Harefield NHS Foundation Trust, London, United Kingdom; j CHU Bordeaux, Department of Cardiovascular Anesthesia and Critical Care, F-33000 Bordeaux, France; k Department of Intensive Cardiac Care, Medical University of Bilaystok, Poland; l German Center for Cardiovascular Research (DZHK), Partner Site Hamburg/Kiel/Lübeck, Hamburg, Germany; m Department of Cardiology, University Heart and Vascular Center Hamburg, University Medical Center Hamburg-Eppendorf, Hamburg, Germany; n Heart Failure and Transplantation Department, Mechanical Circulatory Support and Transplant Department, National Institute of Cardiology, Warsaw, Poland; o Department of Cardiology, Poznan University of Medical Sciences, Poznań, Poland; p Univ. Bordeaux, INSERM, Biologie des Maladies Cardiovasculaires, U1034, F-336000, Pessac, France

**Keywords:** Microaxial flow pump, Older patients, Cardiogenic shock, Mechanical circulatory support, Age

## Abstract

**Background:**

Age represents a critical prognostic factor in cardiogenic shock (CS), yet older patients are underrepresented in randomized trials evaluating temporary mechanical circulatory support (tMCS). We aimed to assess the impact of age on outcomes in CS patients supported with micro-Axial Flow Pump (mAFP) across the age spectrum.

**Methods:**

We analyzed patients with CS treated with mAFP (Impella CP, 5.0 and 5.5) across 11 European centers between 2010 and 2023. Patients were stratified into quartiles by age: Q1 ≤ 52.0 years, Q2 > 52.0–60.0 years, Q3 > 60.0–69.0 years, and Q4 ≥ 69.0 years. The association between age and mortality was assessed using a Fine-Gray competing risks model and restricted cubic spline analysis. The primary endpoint was all-cause mortality at 30 days.

**Results:**

Among 1,043 patients supported with mAFP, the oldest quartile (Q4) was more frequently female, had higher comorbidity burden, and received less intensive support compared to Q1. In the Fine-Gray competing risks model, Q3 and Q4 patients had significantly higher 30-day and 1-year mortality risks (30-day: adjusted sHR 1.66 [95% CI: 1.19–2.34] and 1.87 [1.34–2.62]; 1-year: sHR 1.78 [1.31–2.41] and 2.08 [1.54–2.80]; Ptrend < 0.01 for all), with each additional year of age conferring a 3.2% increase in 30-day mortality risk (adjusted sHR 1.032 [1.017–1.047]). Major left ventricular ejection fraction (LVEF) recovery (≥10 percentage points) declined progressively with age, from 74.5% in Q1 to 41.9% in Q4 (p < 0.01), with each year associated with a 0.40 percentage-point reduction in LVEF improvement.

**Conclusions:**

In this European registry, increasing age was associated with higher mortality and lower observed myocardial recovery among patients with cardiogenic shock supported with mAFP.

## Introduction

Heart failure (HF) and cardiogenic shock (CS) are increasingly prevalent and now represent one of the leading global causes of morbidity and mortality [1,2] Despite advances in pharmacological therapies, immediate reperfusion therapy [[3], [4], [5]] and mechanical circulatory support (MCS), CS remains associated with a poor prognosis, with a 30-day mortality rate of approximately 25–30% [6]. This persistently high mortality is partly explained by the heterogeneity of CS etiologies and clinical phenotypes, each requiring individualized management approaches [7,8]. In this context, the DanGer Shock trial [9] marked a significant advancement, demonstrating that the routine use of a microaxial flow pump (mAFP) in combination with standard care significantly improved 180-day overall survival in acute myocardial infarction–related CS (AMICS) patients, albeit at the cost of an increased risk of adverse events. However, subgroup analyses from DanGer Shock suggested that the survival benefit was not observed in patients older than 77 years [10], and the trial population was limited to AMICS, leaving uncertainty regarding the effectiveness of mAFP in older patients and in CS arising from diverse etiologies.

Age remains among the strongest predictors of outcome, consistently associated with higher mortality across CS severities [11,12]. However, older patients remain underrepresented in randomized clinical trials (RCTs) and are less frequently offered MCS in real-world practices [13]. As populations age, the proportion of older patients presenting with CS is expected to rise sharply, straining healthcare systems worldwide [14]. This underrepresentation in RCTs limits the development of evidence-based, tailored management strategies for this growing population.

Managing CS in older patients is particularly challenging. They often present with more severe forms of CS [15,16], while treatment options are constrained by comorbidities, frailty, and ethical dilemmas regarding the balance between life prolongation and quality of life [14]. These factors often lead to therapeutic restraint and limited use of invasive strategies such as mAFP [17].

Therefore, the aim of this study was to compare the characteristics and outcomes of CS patients supported with mAFP according to age, leveraging data from a large real-world European multicenter registry of CS from diverse etiologies.

## Methods

### Study design and data collection

The IMPACT registry (Clinical-Trials.gov Identifier: NCT06644963) is an international registry of mAFP (Impella CP and Impella 5+ (including Impella 5.0 and 5.5)) that includes data since 2010 from 11 European centers in 5 countries (Supplementary Table S1). The data were collected retrospectively by investigators in each center using an electronic case report form, and consistency tests were performed by data managers. All participating hospitals are large centers experienced in the treatment of CS in general and in the use of mechanical circulatory support devices in particular. Patient and mAFP management, including anticoagulation, screening for bleeding/thrombosis complications and weaning protocol, were at the discretion of each center and per local guidelines. SCAI shock stage was retrospectively adjudicated for each patient based on clinical, hemodynamic, and laboratory data available at the time of mAFP implantation. The analysis followed the Strengthening the Reporting of Observational Studies in Epidemiology (STROBE) guidelines (Supplementary Table S2).

### Population

We analyzed all adult patients (≥18 years) supported with Impella CP and 5+ (5.0 and 5.5) between January 1, 2010, and December 31, 2023. All CS etiologies were included. Patients supported by Impella 2.5 or right-Impella were excluded.

### Outcomes

The primary endpoint was all-cause mortality at 30 days. Secondary endpoints included all-cause mortality at 1 year, cause of death, and left ventricular ejection fraction (LVEF) recovery defined as the change in LVEF between baseline and hospital discharge after device removal, assessed by echocardiography.

### Statistical analysis

Continuous variables were reported as medians and interquartile ranges (IQR). Categorical variables were described as frequencies and percentages. The overall population was divided into quartiles based on age at admission. Continuous variables were compared using the Kruskal-Wallis test and categorical variables were compared using the Pearson chi-square test or the Fisher exact test when appropriate; the resulting values are reported as “p value”. To analyze trends across quartiles, P-trends were computed using the Cochran-Armitage test for binary variables to test for linear trends in proportions, and the Jonckheere-Terpstra test for continuous variables to test for monotonic trends, with both tests assuming independent observations and ordered groups (age quartiles). Additionally, a Fine-Gray competing risks regression model was used to assess the association between all-cause mortality and age, treating heart transplantation (HTx), permanent ventricular assist device (VAD) implantation, and escalation to VA-ECMO as competing events, adjusted with the following covariates: sex, body mass index, history of ischemic heart disease, atrial fibrillation, HF, and chronic kidney disease; comorbidities (chronic obstructive pulmonary disease, dyslipidemia, diabetes mellitus, stroke); type of mAFP (CP or 5+); cardiac arrest; CS etiology; baseline LVEF measured at mAFP implantation, creatinine, and lactate levels; use of norepinephrine, dobutamine, or epinephrine at implantation; ventilation strategy; SCAI stage; and participating center. Results are presented as adjusted subdistribution hazard ratios (sHRs) with 95% confidence intervals (CIs). These variables were selected based on clinical and pathophysiological relevance, in accordance with the Prognosis Research Strategy (PROGRESS) guidelines [18], which advocate for a hypothesis-driven, a priori selection of covariates rather than reliance on univariable statistical associations. For the purposes of multivariable analyses, missing values were addressed using multiple imputations with chained equations generating 10 imputed datasets. Multicollinearity was evaluated by calculating variance inflation factors, with value >5 indicating potential multicollinearity concerns. As a sensitivity analysis, patients were additionally dichotomized into two age groups (Q1 + Q2, age ≤ 60 years vs Q3 + Q4, age > 60 years) and the Fine-Gray competing risks model was repeated accordingly. Multicollinearity was reassessed using variance inflation factors. To account for heterogeneity in clinical practices across centers, a sensitivity analysis was performed using a mixed-effects Cox model incorporating country as a fixed effect and center as a random effect with a random slope for age, adjusted for the same covariates as the primary analysis, treating HTx, permanent VAD implantation, and VA-ECMO escalation as censoring events, with results presented as adjusted hazard ratios (HRs). In addition, a Fine-Gray competing risks model with restricted cubic spline (RCS) functions with 3-knots positioned at the 10th, 50th, and 90th percentiles of the age distribution was performed in the overall population to assess the shape of the associations between age (as a continuous measure) and 30-day all-cause mortality. Potential nonlinearity was evaluated using a likelihood ratio test, comparing the model with only a linear term to the model including both linear and cubic spline terms. To assess the robustness of this relationship, two pre-specified sensitivity analyses were performed using the same restricted cubic spline approach: one restricted to patients with an mAFP support duration greater than one day, and one restricted to patients in the intermediate age quartiles (Q2 and Q3), thereby excluding extreme ages. Eventually, based on the gaps identified in the literature and pathophysiological considerations [12,13], an exploratory assessment of LVEF improvement between baseline and discharge was performed, categorizing patients into four groups: deterioration (≤5%), stable (±5%), moderate improvement (+5–9%), and major improvement (≥10%). The association between age and LVEF recovery was assessed using linear regression models, with the change in LVEF as the dependent variable and age as a continuous predictor, adjusted for the same baseline covariates used in the survival analysis to control for potential confounding factors, particularly CS etiology.

All tests were two-tailed. A value of p ≤ 0.05 was accepted as statistically significant. Analyses were performed using R software [version 4.3.2].

### Ethics

The study was conducted in accordance with the Declaration of Helsinki and was approved by local ethics committees and institutional review committees (IRB Accreditation number: 198711). According to the main ethics committee, written consent was waived because of the observational and retrospective design of the study and only completely anonymized data were collected and analyzed.

## Results

### Baseline patient characteristics

Overall, 1,043 patients with CS supported with mAFP were included from 11 European centres, whose baseline characteristics are depicted in Table 1. Participants were stratified into four quartiles according to age at admission: Quartile 1 (Q1) ≤52.0 years, Quartile 2 (Q2) >52.0 to 60.0 years, Quartile 3 (Q3) >60.0 to 69.0 years, and Quartile 4 (Q4) ≥69.0 years.

**Table 1:** Baseline characteristics according to age quartiles.

	Missing values, n (%)	Quartile 1 (≤52 years) (n = 273)	Quartile 2 (>52–60 years) (n = 249)	Quartile 3 (>60–69 years) (n = 264)	Quartile 4 (>69 years) (n = 257)	p value	P_trend_
Male sex, n (%)	0 (0.0)	199 (72.9)	203 (81.5)	219 (83.0)	175 (68.1)	< 0.01	0.32
BMI, kg/m^2^, median (IQR)	97 (9.3)	24.3 (22.0–28.6)	26.1 (23.6–29.4)	26.1 (24.1–29.2)	26.0 (23.4–28.9)	< 0.01	< 0.01
History of ischemic cardiomyopathy, n (%)	14 (1.3)	45 (16.5)	73 (29.4)	99 (38.2)	91 (36.4)	< 0.01	< 0.01
Atrial fibrillation, n (%)	8 (0.8)	18 (6.6)	26 (10.5)	35 (13.4)	50 (19.7)	< 0.01	< 0.01
History of heart failure, n (%)	18 (1.7)	55 (20.0)	46 (18.7)	56 (21.6)	66 (26.6)	0.16	0.06
ICD, n (%)	385 (36.9)	16 (7.5)	20 (10.8)	13 (7.6)	8 (9.1)	0.63	0.84
Comorbidities, n (%)							
COPD	13 (1.2)	3 (1.1)	10 (4.1)	14 (5.4)	31 (12.3)	< 0.01	< 0.01
Dyslipidemia	46 (4.4)	52 (19.3)	71 (29.2)	102 (40.8)	126 (53.6)	< 0.01	< 0.01
Diabetes mellitus	12 (1.2)	32 (11.8)	44 (17.9)	84 (32.3)	87 (34.4)	< 0.01	< 0.01
Chronic kidney disease (eGFR ≤60 mL/min)	20 (1.9)	13 (4.8)	22 (8.9)	30 (11.7)	22 (8.8)	0.04	0.049
Stroke	15 (1.4)	16 (5.9)	13 (5.3)	18 (6.9)	13 (5.2)	0.82	0.97
Peripheral artery disease	392 (37.6)	8 (3.7)	13 (7.1)	21 (12.4)	5 (5.9)	0.01	0.04
Vascular surgery	350 (33.6)	6 (0.9)	8 (4.1)	6 (3.2)	4 (4.4)	0.81	0.58
Cirrhosis	350 (33.6)	2 (0.9)	5 (2.6)	1 (0.5)	4 (4.4)	0.07	0.22
Microaxial flow pump device, n (%)	0 (0.0)					0.01	
Impella CP		176 (64.5)	158 (63.5)	184 (69.7)	232 (90.3)		< 0.01
Impella 5+		97 (35.5)	91 (36.5)	80 (30.3)	25 (9.7)		< 0.01
Impella total length, days, median (IQR)	9 (0.9)	5.0 (3.0–10.0)	6.0 (2.0–10.0)	4.0 (2.0–8.0)	1.0 (0.0–4.0)	< 0.01	< 0.01
Pre-assist cardiac arrest, n (%)	39 (3.7)	97 (36.5)	77 (32.1)	75 (30.7)	62 (24.4)	0.03	< 0.01
Shock etiology, n (%)							
Acute myocardial infarction	1 (0.1)	123 (45.2)	168 (67.5)	185 (70.1)	158 (61.5)	< 0.01	< 0.01
Electrical storm	0 (0.0)	19 (7.0)	16 (6.4)	30 (11.4)	10 (3.9)	0.01	0.57
Post-cardiotomy	0 (0.0)	19 (7.0)	16 (6.4)	16 (6.1)	15 (5.8)	0.96	0.58
Fulminant myocarditis	0 (0.0)	35 (12.8)	3 (1.2)	3 (1.1)	1 (0.4)	< 0.01	< 0.01
Acute-on-chronic heart failure	0 (0.0)	49 (17.9)	28 (11.2)	24 (9.1)	9 (3.5)	< 0.01	< 0.01
Acute valvular heart disease	0 (0.0)	4 (1.5)	9 (3.6)	3 (1.1)	4 (1.6)	0.21	0.59
Hemodynamic assessment at Impella placement							
LVEF, %, median (IQR)	183 (17.5)	15.0 (10.0–21.8)	20.0 (15.0–30.0)	20.0 (15.0–28.0)	25.0 (15.0–30.0)	0.08	< 0.01
Norepinephrine, n (%)	36 (3.5)	179 (69.1)	162 (67.2)	168 (66.4)	128 (50.4)	< 0.01	< 0.01
Dobutamine, n (%)	31 (3.0)	130 (50.0)	124 (50.8)	115 (45.3)	70 (27.6)	< 0.01	< 0.01
Epinephrine, n (%)	31 (3.0)	81 (31.2)	76 (31.1)	89 (35.0)	109 (42.9)	0.02	< 0.01
Lactates, mmol/L, median (IQR)	116 (11.1)	4.0 (2.0–7.4)	3.7 (1.8–7.7)	3.5 (2.0–7.8)	4.2 (2.1–8.3)	0.62	0.43
Respiratory support at Impella placement, n (%)	23 (2.2)					< 0.01	
Invasive		172 (64.9)	173 (71.2)	165 (64.2)	132 (51.8)		< 0.01
Non-invasive		93 (35.1)	70 (28.8)	92 (35.8)	123 (48.2)		< 0.01
Biology at Impella placement, median (IQR)							
Creatinine, μmol/L	41 (3.9)	109.0 (78.5–144.6)	123.0 (90.0–158.0)	117.0 (90.5–168.0)	113.0 (91.0–155.0)	0.01	0.04
ASAT, U/L	269 (25.8)	348.0 (92.3–874.5)	215.5 (73.5–817.0)	301.0 (98.0–721.0)	320.0 (115.0–824.0)	0.37	0.71
ALAT, U/L	149 (14.3)	128.0 (58.0–311.0)	111.5 (45.0–290.8)	116.0 (59.0–307.0)	89.0 (39.5–190.0)	0.02	0.02
Bilirubin, μmol/L	387 (37.1)	16.0 (9.0–29.0)	16.8 (9.0–25.5)	15.0 (10.2–25.0)	14.0 (9.5–23.0)	0.67	0.37
Prothrombin ratio, %	398 (38.2)	70.0 (49.0–91.5)	69.5 (50.3–89.8)	74.0 (56.0–90.5)	68.0 (50.0–81.0)	0.2	0.78
Platelets, G/L	35 (3.4)	191.0 (127.0–265.0)	195.0 (139.0–267.0)	205.0 (146.0–263.5)	214.0 (155.8–275.3)	0.06	< 0.01
Glasgow coma scale at Impella placement, n (%)	403 (38.6)					0.49	
15		110 (53.7)	86 (47.3)	93 (56.4)	48 (54.5)		0.57
7–14		4 (2.0)	9 (4.9)	6 (3.6)	3 (3.4)		0.49
≤6		91 (44.4)	87 (47.8)	66 (40.0)	37 (42.0)		0.41
RRT, n (%)	58 (5.6)	100 (38.0)	101 (42.4)	86 (36.1) (n = 238)	63 (25.6)	< 0.01	< 0.01
SCAI, n (%)	0 (0.0)					< 0.01	
B		7 (2.6)	13 (5.2)	11 (4.2)	26 (10.1)		< 0.01
C		56 (20.5)	52 (20.9)	80 (30.3)	56 (21.8)		0.25
D		100 (36.6)	95 (38.2)	79 (29.9)	88 (34.2)		0.24
E		110 (40.3)	89 (35.7)	94 (35.6)	87 (33.9)		0.14

ALAT, alanine aminotransferase; ASAT, aspartate aminotransferase; BMI, body mass index; COPD, chronic obstructive pulmonary disease; ECMO-VA, extracorporeal membrane oxygenation-venoarterial; eGFR, estimated glomerular filtration rate; ICD, implantable cardioverter-defibrillator; IQR, interquartile range; LVEF, left ventricular ejection fraction; RRT, renal replacement therapy.

Compared with younger individuals (Q1), older patients (Q4) were more frequently female (31.9% vs 27.1%, p < 0.01), had a higher body mass index (26.0 vs 24.3 kg/m², p < 0.01; Ptrend < 0.01), and showed higher rates of ischemic cardiomyopathy (36.4% vs 16.5%, p < 0.01; Ptrend < 0.01) and atrial fibrillation (19.7% vs 6.6%, p < 0.01; Ptrend < 0.01). Similarly, the burden of comorbidities including chronic obstructive pulmonary disease, dyslipidaemia, diabetes and chronic kidney disease increased with age, with all showing a significant trend.

Impella CP was more frequently used in older patients (rising from 64.5% in Q1 to 90.3% in Q4, p < 0.01), whereas Impella 5+ was more often chosen for younger individuals (35.5% in Q1 to 9.7% in Q4, p < 0.01), both with significant Ptrend. The prevalence of AMICS increased with age (Ptrend < 0.01), while fulminant myocarditis and acute-on-chronic HF were more common among the younger groups.

At implantation, older patients had higher LVEF (25.0% vs 15.0%, Ptrend < 0.01) and received lower rates of norepinephrine (50.4% vs 69.1%, p < 0.01), dobutamine (27.6% vs 50.0%, p < 0.01), and invasive mechanical ventilation (51.8% vs 64.9%, Ptrend < 0.01). The use or renal replacement therapy decreased with age (25.6% in Q4 vs 38.0% in Q1, Ptrend < 0.01). Finally, SCAI stage D or E was present in most patients across all age quartiles (77.1%, 73.9%, 65.5%, and 68.1% in Q1 to Q4, respectively), with a higher proportion of SCAI stage B in older patients (p < 0.01).

### Short and long-term outcomes

**Fig. 1:**
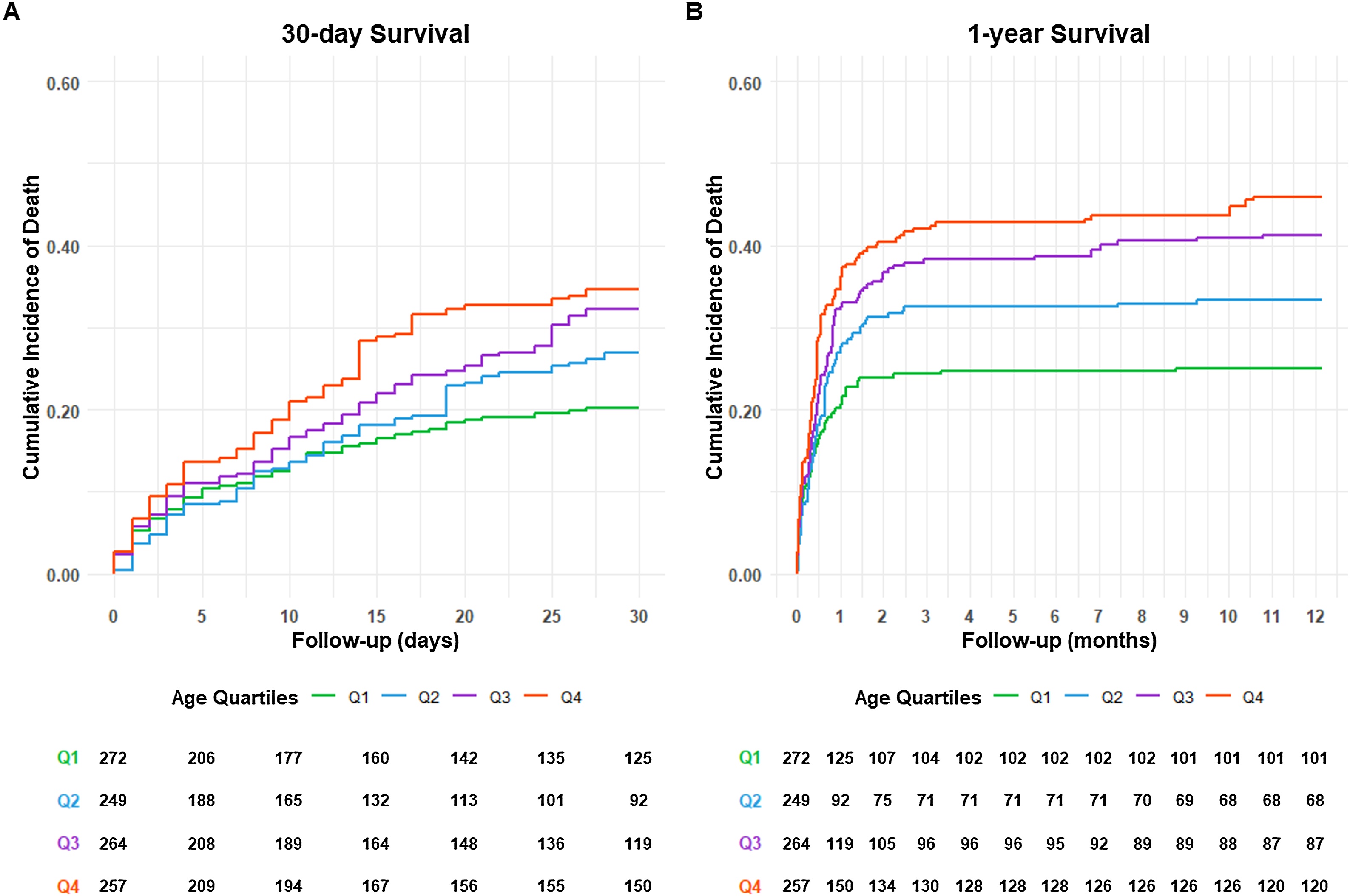
Overall Survival According to Age Quartiles in Patients with Cardiogenic Shock Treated with mAFP.

In the Fine-Gray competing risks model, a significant gradient of 30-day all-cause mortality was observed across age quartiles. Compared with Q1, patients in Q3 and Q4 had significantly higher 30-day mortality risks (adjusted sHR 1.66 [95% CI: 1.19–2.34], p < 0.01 and 1.87 [95% CI: 1.34–2.62], p < 0.01, respectively; Ptrend < 0.01). A similar pattern was observed at 1 year, with adjusted sHRs of 1.78 (95% CI: 1.31–2.41, p < 0.01) and 2.08 (95% CI: 1.54–2.80, p < 0.01) for Q3 and Q4, respectively (Ptrend < 0.01) (Fig. 1, Table 2). The incidence of competing events, including heart transplantation, permanent VAD implantation, and VA-ECMO escalation, were all significantly lower in older patients compared with younger ones (Ptrend < 0.01 for all). In the binary age group sensitivity analysis, older patients (Q3 + Q4, age > 60 years) had significantly higher 30-day and 1-year mortality compared to younger patients (Q1 + Q2, age ≤ 60 years) (adjusted sHR 1.80 [95% CI 1.23–2.55], p < 0.01 and 1.72 [95% CI: 1.27–2.33], p < 0.01, respectively) (Supplementary Fig. S1).

**Table 2:** Fine-Gray Competing Risks Models Evaluating 30-Day and 1-Year All-Cause Mortality and Competing Events According to Age Quartiles.

			30 days			1 year		
		Number of patients	Events, n (%)	Adjusted sHR (95% CI), p value	P_trend_	Events, n (%)	Adjusted sHR (95% CI), p value	P_trend_
All-cause mortality	Q1	273	55 (20.1)	Ref		68 (24.9)	Ref	
	Q2	249	67 (26.9)	1.35 (0.94–1.92), 0.10		83 (33.3)	1.38 (0.99–1.90), 0.051	
	Q3	264	85 (32.2)	1.66 (1.19–2.34), < 0.01	< 0.01	109 (41.3)	1.78 (1.31–2.41), < 0.01	< 0.01
	Q4	257	89 (34.6)	1.87 (1.34–2.62), < 0.01		118 (45.9)	2.08 (1.54–2.80), < 0.01	
Heart transplantation	Q1	273	25 (9.2)	Ref		28 (10.3)	Ref	
	Q2	249	18 (7.2)	0.78 (0.43–1.43), 0.42		20 (8.0)	0.77 (0.44–1.37), 0.38	
	Q3	264	5 (1.9)	0.20 (0.08 – 0.52), < 0.01	< 0.01	7 (2.7)	0.25 (0.11 – 0.57), < 0.01	< 0.01
	Q4	257	0 (0.0)	NA		0 (0.0)	NA	
Ventricular assist device	Q1	273	30 (11.0)	Ref		38 (13.9)	Ref	
	Q2	249	33 (13.3)	1.21 (0.74–1.98), 0.45		38 (15.3)	1.10 (0.70–1.73), 0.68	
	Q3	264	32 (12.1)	1.10 (0.67–1.81), 0.71	< 0.01	38 (14.4)	1.03 (0.66–1.62), 0.89	< 0.01
	Q4	257	3 (1.2)	0.10 (0.03 – 0.33), < 0.01		4 (1.6)	0.10 (0.04 – 0.29), < 0.01	
ECMO	Q1	273	37 (13.6)	Ref		37 (13.6)	Ref	
	Q2	249	39 (15.7)	1.17 (0.74–1.84), 0.49		39 (15.7)	1.17 (0.74–1.84), 0.49	
	Q3	264	23 (8.7)	0.63 (0.38–1.06), 0.08	< 0.01	23 (8.7)	0.63 (0.38–1.06), 0.08	< 0.01
	Q4	257	15 (5.8)	0.42 (0.23 – 0.76), < 0.01		15 (5.8)	0.42 (0.23 – 0.76), < 0.01	

sHR, subdistribution hazard ratio; CI, confidence interval; ECMO, extracorporeal membrane oxygenation; VAD, ventricular assist device.

In the mixed-effects Cox sensitivity analysis, results were consistent with the primary analysis, with significantly higher 30-day and 1-year mortality risks in Q3 and Q4 compared to Q1 (30-day: adjusted HR 1.64 [95% CI: 1.16–2.32] and 2.06 [95% CI: 1.43–2.96]; 1-year: adjusted HR 1.70 [95% CI: 1.25–2.32] and 2.00 [95% CI: 1.45–2.76], respectively; all p < 0.01), with no evidence of center-level heterogeneity in the age-mortality relationship (random slope variance for age < 0.01 at 30 days and 1 year).

No evidence of problematic multicollinearity was detected, with all variance inflation factors <2.0 across covariates for all analyses.

The distribution of causes of death significantly varied across age quartiles. While younger patients (Q1) most frequently died from anoxic brain injury (20.0%), older patients (Q4) exhibited a predominance of cardiovascular-related deaths (41.6%) (Ptrend < 0.01) (Supplementary Table S3). Detailed mortality rates by age quartile and CS etiology are presented in Supplementary Table S4.

**Fig. 2:**
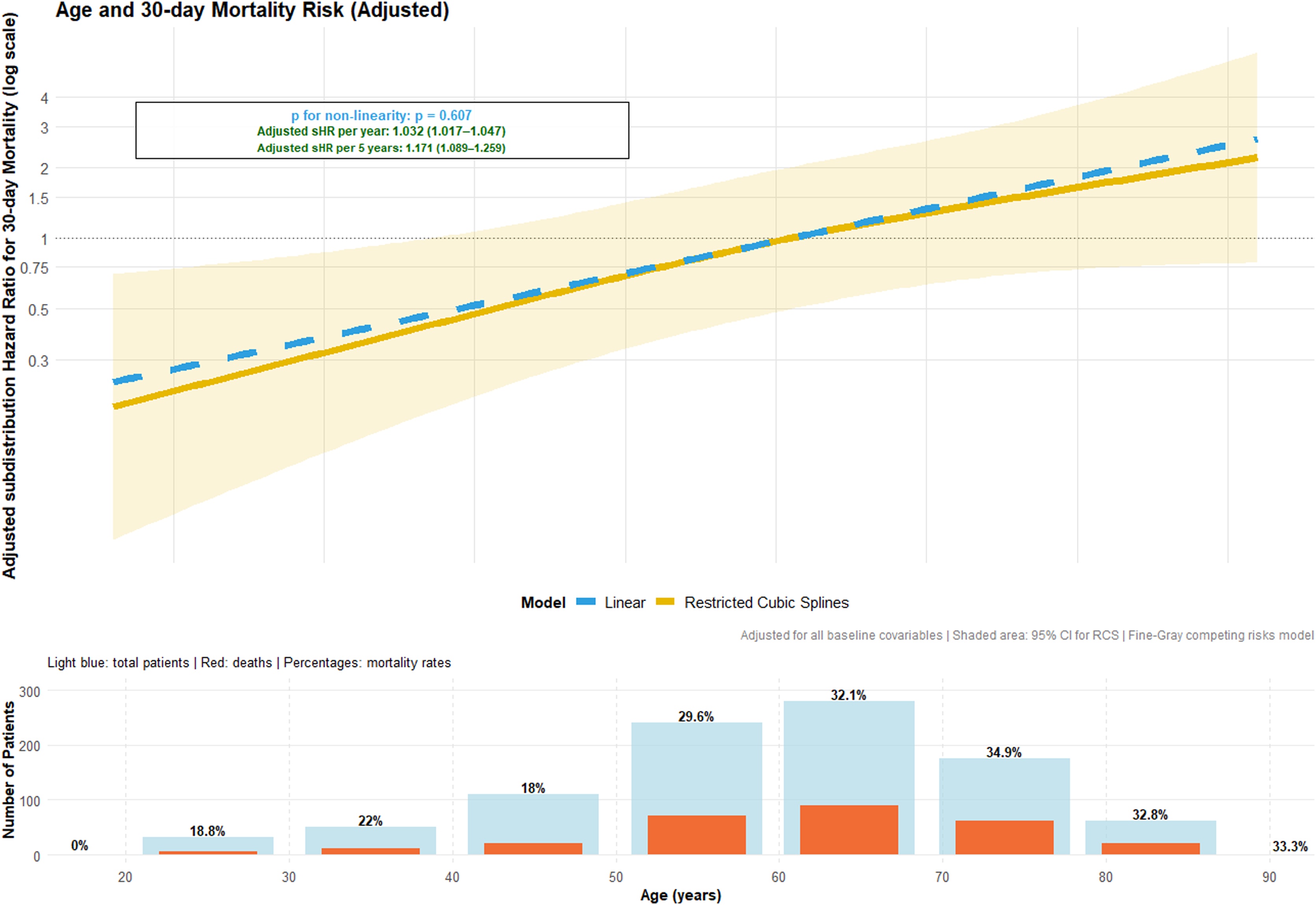
Relationship Between Age and Adjusted 30-Day Mortality Risk Using Restricted Cubic Splines. RCS and linear models yield virtually identical results, suggesting the linear relationship between age and mortality. aHR, adjusted hazard ratio.

The relationship between age and 30-day all-cause mortality risk showed no evidence of non-linearity by spline analysis, with p-value for non-linearity of 0.61 (Fig. 2). After adjustment for all baseline covariables, each year increase in age was associated with a 3.2% increase in 30-day mortality risk (adjusted sHR 1.032, 95% CI: 1.017–1.047), translating to a 17.1% increase per 5-year increment (adjusted sHR 1.171, 95% CI: 1.089–1.259). In sensitivity analyses, he association between age and 30-day mortality remained consistent when restricted to patients with mAFP support duration greater than one day (adjusted sHR per year: 1.039 [95% CI: 1.020–1.057]; per 5 years: 1.208 [95% CI: 1.106–1.321]) and when restricted to intermediate age quartiles (adjusted sHR per year: 1.052 [95% CI: 1.006–1.100]; per 5 years: 1.291 [95% CI: 1.032–1.614]) (Supplementary Fig. S2).

### Myocardial recovery

**Fig. 3:**
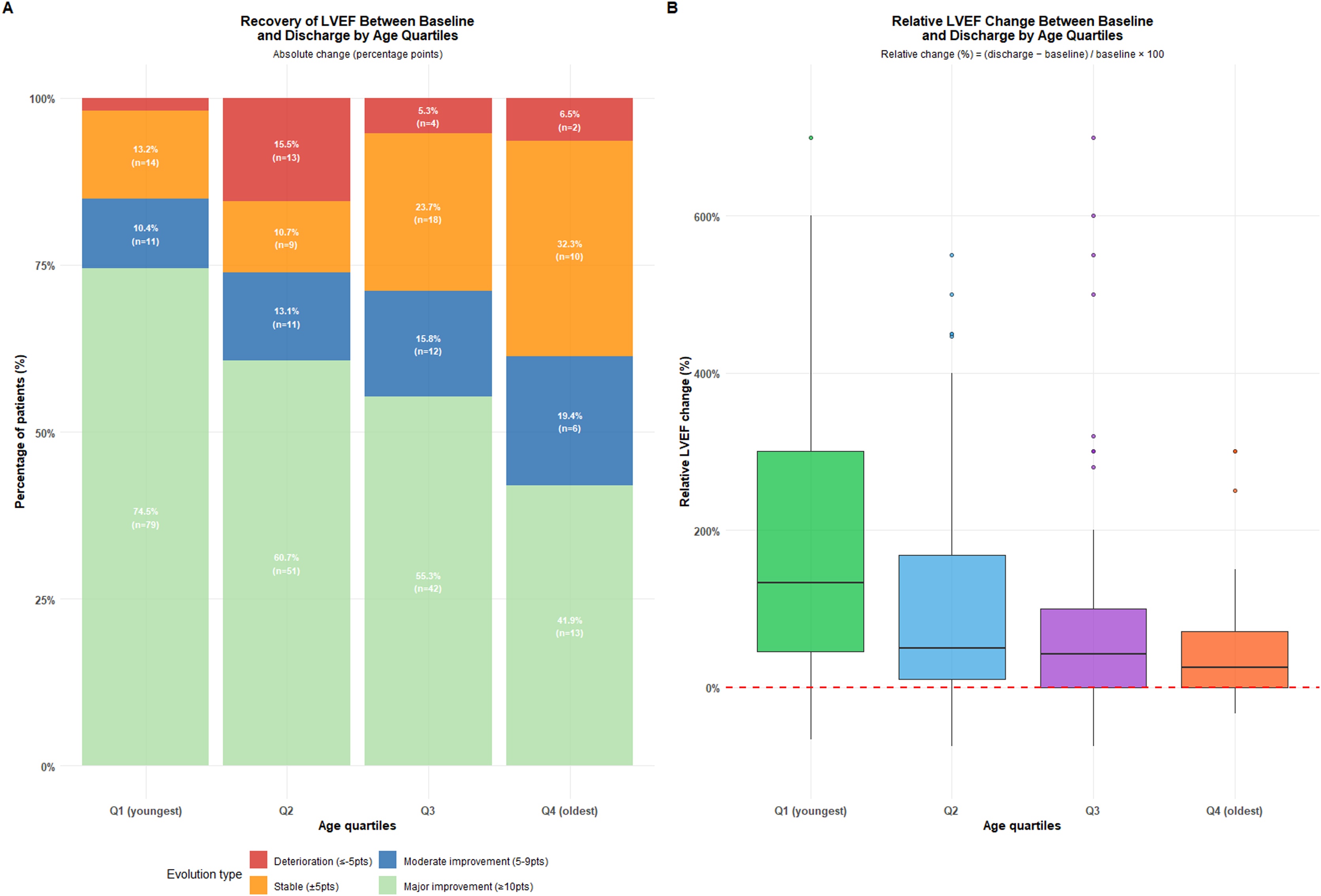
Left Ventricular Ejection Fraction Recovery Between Baseline and Discharge by Age Quartiles. LVEF, left ventricular ejection fraction.

A significant inverse relationship was observed between age and LVEF recovery capacity, with younger patients demonstrating markedly superior myocardial functional improvement compared to older patients (p < 0.01) (Fig. 3). The youngest quartile (Q1) exhibited the highest proportion of major LVEF improvement (≥10 percentage points) at 74.5%, which progressively declined across age groups to reach only 41.9% in the oldest quartile (Q4). Conversely, Q1 less often had a deterioration in LVEF compared to Q2-Q4. After adjustment for baseline characteristics, CS etiology and severity markers, linear regression analysis confirmed that each year of advancing age was independently associated with a 0.40-percentage points (95% CI 0.29−0.49) reduction in LVEF recovery (p < 0.01), translating to 2.0-percentage points (95% CI 1.45–2.45) decrease per 5-year increment. A similar pattern was observed when using relative LVEF change as the outcome, with a progressive decline in relative myocardial recovery across age quartiles (Q1: median +133%; Q2: +53%; Q3: +42%; Q4: +25%; Ptrend < 0.01).

## Discussion

To date, this is the largest multicenter international study evaluating the characteristics and outcomes of CS patients supported with mAFP across the age spectrum. Our main findings are as follows: [1] after multivariable adjustment, increasing age was associated with higher short- and long-term mortality in patients with CS supported with mAFP, although this gradient was not apparent in unadjusted analyses [2]; in adjusted analyses, advancing age was associated with lower observed rates of left ventricular functional recovery, likely reflecting a combination of age-related factors, differences in CS management and difference in underlying shock etiology [3] older patients were treated with less intensive support strategies, suggesting a more selective and restrained use of mAFP in this population.

Age is among the most well-established and uncontested risk factors for mortality in patients with CS, consistently reported across all large-scale studies on the subject [15,16]. Our results reinforce this observation, showing that each 5-year increase in age is associated with a 17% rise in 30-day mortality. Importantly, while baseline characteristics differed significantly across age groups, with older patients paradoxically presenting less severe illness profiles, this differential severity masked the true age effect in unadjusted analyses. Multivariable adjustment revealed the gradual detrimental effect of increasing age by controlling for these confounding baseline differences. This align with the DanGer Shock trial, which reported substantially higher mortality among older patients with AMICS and may derive less benefit from routine treatment with mAFP compared to younger patients [19]. Importantly, although age is undeniably a prognostic factor, this does not imply that older patients should be systematically excluded from mAFP support. Rather, it highlights the need for carefully defined selection criteria that incorporate both the current or anticipated severity of shock and a comprehensive evaluation of physiological and cognitive reserve [17]. The excess mortality observed in older patients likely results from a combination of age-related physiological vulnerabilities: reduced hemodynamic adaptability, increased frailty, polymedication, multimorbidity, and altered pharmacokinetics [20]. In our cohort, older patients presented more frequently with comorbid conditions such as chronic obstructive pulmonary disease, dyslipidemia, diabetes, chronic kidney disease, and prior stroke, although they were less frequently treated with norepinephrine, dobutamine, invasive ventilation, or renal replacement therapy, suggesting a degree of therapeutic self-restriction in anticipation of poor outcomes. On the other hand, mAFP support carries a significant risk of device-related complications, including acute kidney injury, hemocompatibility-related adverse events, and vascular complications, particularly in the presence of tortuous, small-caliber, and calcified arteries, which poses a major clinical challenge, as older patients are both more susceptible to CS-related complications [21], and less likely to tolerate device-related adverse effects due to reduced physiological reserve [22]. As a result, weighing the risk–benefit ratio of mAFP therapy in this population is particularly complex. However, in our study, we did not observe a higher rate of device-related complications, such as acute kidney injury or bleeding, in older patients, consistent with recent findings from the DanGer Shock trial [19]. Several factors may explain this apparent paradox. First, early mortality in the oldest quartile may have introduced a survival bias: older patients may have died before developing complications, whereas younger patients, surviving longer, had more time for adverse events to occur. Second, treatment escalation (e.g., to additional MCS) was more commonly pursued in younger individuals, which may partially explain the unexpected finding of a higher incidence of bleeding complications in the youngest subgroup. Third, mAFP may have been used more selectively in older adults, with implicit bias toward patients with more favorable profiles, fewer comorbidities, preserved renal function, and less severe multiorgan failure, thereby attenuating complication rates in this group. Taken together, these effects may have balanced the overall complication rate across age groups, especially given the inherent risk of adverse events associated with mechanical circulatory support.

Furthermore, the markedly short median support duration observed in the oldest quartile likely reflects the coexistence of three complementary phenomena: early death with rapid device removal in the context of futility, limited escalation to additional MCS when mAFP alone proved insufficient, and early successful weaning in a subset of carefully selected older patients implanted for brief hemodynamic stabilization. This pattern is consistent with a more selective and restrained implantation strategy in this age group.

Myocardial recovery represents another critical dimension where age significantly impacts short but also long-term outcomes in CS patients. Our findings demonstrate a strong inverse relationship between advancing age and cardiac functional improvement, with younger patients achieving major LVEF recovery (≥10 percentage points) in 74.5% of cases compared to only 41.9% in the oldest quartile. CS encompasses heterogeneous etiologies with distinct prognostic trajectories. Younger patients more often presented with potentially reversible etiologies, whereas ischemic shock predominated in older patients, which may partly account for age-related differences in mortality and left ventricular recovery. From a mechanistic perspective, several biological processes may explain this attenuated recovery potential in the aging myocardium. Aging is associated with impaired mitochondrial bioenergetics, altered calcium handling, progressive myocardial fibrosis, and heightened inflammatory responses [[23], [24], [25]]. These changes amplify ischemia–reperfusion injury and blunt the beneficial effects of unloading, thereby limiting the durable myocardial recovery. These observations align with well-established clinical findings in data regarding left VAD, where advanced age consistently emerges as a negative predictor of myocardial recovery and successful bridge-to-recovery strategies [26]. Studies in VAD patients have demonstrated that younger age is strongly associated with higher rates of cardiac functional improvement and successful device weaning, with recovery rates declining substantially beyond 40–50 years old [27]. Given the excellent contemporary outcomes of VAD implantation even in well-selected septuagenarians [28], our results underscore the importance of early VAD evaluation in this subgroup. Two clinical scenarios may be considered: first, patients who fail to recover, in whom early VAD implantation should be considered during the acute phase; and second, patients successfully weaned from mAFP but who remain at high risk of subsequent deterioration. In this latter group, timely reassessment in an expert center is essential to avoid missing the optimal window for implantation.

Taken together, these observations suggest that management of older patients with CS may benefit from a comprehensive clinical assessment that extends beyond chronological age alone. In line with current clinical practice and guideline recommendations, a multidisciplinary approach may help support individualized decision-making in this complex population [29].

## Limitations

The main limitation of our study lies in its retrospective design, which inherently carries a risk of selection bias, further amplified by the fact that all data were derived from high-volume expert centers in Europe, potentially limiting the generalizability of our findings to other settings. Additionally, practice heterogeneity across centers, particularly regarding anticoagulation protocols and rotor-speed management, may have influenced outcomes. Another important limitation is the lack of data concerning geriatric assessment of general and functional status, including frailty criteria or comprehensive comorbidity indices such as the Charlson Comorbidity Index. This absence precluded stratified analyses by frailty level, which could have helped identify subgroups of older patients more likely to benefit from aggressive support strategies. Future studies should aim to incorporate these variables to guide more personalized and appropriate care. Moreover, while our study focused on mortality and early myocardial recovery, data regarding long-term myocardial recovery and the use of guideline-directed medical therapy (GDMT) were not available. Although our models were adjusted for CS etiology, residual confounding cannot be excluded and likely contributes to the observed age-related differences in myocardial recovery. Finally, the absence of a non–mAFP-supported control group restricts the ability to directly compare outcomes with alternative support strategies, and no data were available regarding the transition to palliative care, which remains a key component of management in advanced-age patients with poor prognostic outlooks.

## Conclusion

In this large multicenter retrospective registry of patients with cardiogenic shock supported with mAFP, increasing age was associated with higher short- and long-term mortality and with lower observed left ventricular functional recovery. These associations persisted after multivariable adjustment but must be interpreted cautiously given the observational design and the possibility of residual confounding.

These results describe age-related patterns in contemporary clinical practice and illustrate the heterogeneity of older patients with cardiogenic shock. They should be considered exploratory and hypothesis-generating, and they highlight the need for prospective studies to better characterize patient profiles and to evaluate management strategies across the age spectrum.

## Funding source

None.

## Declaration of competing interest

AU, BS, PG, and CD report lectures fees from Abiomed.
