# Supplementary material for: Age-associated outcomes in cardiogenic shock supported with microaxial flow pumps
Source: Ann Intensive Care. 2026 Jun 24;16:100104. doi: 10.1016/j.aicoj.2026.100104 (PMC13330548; doi:10.1016/j.aicoj.2026.100104)
Supplement: Supplementary file 1 [file mmc1.docx]

**Supplementary Material — Description of Microaxial Flow Pumps (mAFP)**

Microaxial flow pumps (mAFP) are catheter-based temporary mechanical circulatory support devices designed to provide active hemodynamic support in patients with cardiogenic shock. In the present study, three Impella devices were used: the Impella CP, Impella 5.0, and Impella 5.5 (Abiomed, Danvers, MA, USA).

These devices consist of a miniaturized axial flow pump mounted on a pigtail catheter, inserted retrograde across the aortic valve, typically via femoral or axillary arterial access, and positioned with the inlet in the left ventricle and the outlet in the ascending aorta. By continuously aspirating blood from the left ventricle and ejecting it into the ascending aorta, mAFP provide direct left ventricular unloading while simultaneously augmenting cardiac output and mean arterial pressure. This dual mechanism is of pathophysiological relevance in cardiogenic shock: by reducing left ventricular end-diastolic pressure and wall stress, mAFP decrease myocardial oxygen consumption while increasing coronary perfusion pressure, thereby potentially creating favorable conditions for myocardial recovery. Furthermore, the reduction in left ventricular filling pressures leads to a decrease in pulmonary congestion, which may improve right ventricular afterload and overall cardiopulmonary hemodynamics. Unlike intra-aortic balloon pumps, which provide passive diastolic augmentation, mAFP deliver active, flow-dependent support, independent of cardiac rhythm, making them effective even in the context of severe systolic dysfunction or arrhythmias. The degree of hemodynamic benefit is directly proportional to the rotational speed of the pump (P-level), which can be titrated at the bedside according to the patient's hemodynamic response and tolerance.

The three Impella devices differ primarily in their flow capacity and access site:

- **Impella CP** delivers up to 3.5 L/min of cardiac output support and is inserted via femoral access (14F sheath), making it the most widely used percutaneous option in the acute setting.
- **Impella 5.0** delivers up to 5.0 L/min and requires surgical cutdown, typically via the axillary or femoral artery, allowing for more durable support and patient mobilization.
- **Impella 5.5** delivers up to 5.5 L/min via axillary access and represents the highest-flow option currently available, particularly suited for patients requiring prolonged or more intensive hemodynamic support.

In clinical practice, mAFP are positioned under fluoroscopic and echocardiographic guidance. Correct positioning is critical: the inlet port must remain within the left ventricle, at least 3.5-4 cm below the aortic valve plane, to ensure optimal flow and avoid suction events or device migration. Hemodynamic monitoring is performed continuously via the device console, which displays a real-time placement signal and flow metrics; and by echocardiography (at least twice a day in normal operation and more often in case of alarms or malfunction).

| **Center** | **Co-investigators** |
| --- | --- |
| Department of Anesthesiology and Critical Care Medicine, Arnaud de Villeneuve Hospital, CHU Montpellier | Ughetto Aurore, MD  Gaudard Philippe MD, PhD  Chelvi Sendin Emma MD  Chouaiki-perrois Arthur MD |
| Intensive Cardiac Care Unit, Cardiology department, Rangueil University Hospital, Toulouse, France | Delmas Clément, MD, PhD,  Cherbi Miloud, MD  Bouisset Frederic, MD  Tassain Chloe  Vardon Fanny MD, PhD |
| CHU Bordeaux, department of cardiovascular anesthesia and critical care, CHU de Bordeaux, France | Beurton Antoine, MD  Imbault Julien MD,  De Cuniac Marie MD  Herion François-Xavier MD  Ouattara Alexandre MD, PhD |
| Department of Cardiology, University of Rennes, CHU Rennes, France | Leurent Guillaume, MD  Leo Lemarchand, MD  Erwan Flecher MD, PhD  Nesseler Nicolas, MD,PhD  Alexandre Mansour, MD, PhD |
| Assistance Publique-Hôpitaux de Marseille, Department of Cardiology, Hôpital Nord, Marseille, France | Bonello Laurent MD, PhD  Hadrien Pichené, MD |
| Department of Critical Care, Harefield Hospital, Royal Brompton and Harefield NHS Foundation Trust, London, UK | Hurtado Ana, MD PhD,  Otero Escudero Macarena, MD  Gonzalez Lastra Sara, MD,  Vandenbriele Christophe MD, PhD  Panoulas Vasileios MD, PhD |
| Department of Cardiology, University Heart and Vascular Center Hamburg, Germany | Schrage Benedikt, MD, PhD  Beer Benedikt MD, PhD  Sundermeyer Jonas, MD  Angela Dettling MD |
| Department of Cardiology, Inselspital, Bern, Switzerland | Fürholz Monika, MD, PhD  Jacomet Matias MD  Lukas Hunziker, MD, PhD |
| Institute for Heart Diseases, Wroclaw medical University, Wroclaw, Poland | Kuliczkowski Wiktor, MD, PhD  Blaziak Mikolaj, MD |
| Heart Failure and Transplantology Department, Mechanical Circulatory Support and Transplant Department, National Institute of Cardiology, Warsaw, Poland | Zaleska Kociecka Marta MD PhD  Tycinska Agnieszka MD, PhD  Konopka Anna MD, PhD |
| Department of Cardiology, Poznan University of Medical Sciences, Poznań, Poland | Klotzka Aneta, MD PhD |

**Supplementary Table 1**: IMPACT co-investigators, listed alongside their centers.

STROBE Statement—checklist of items that should be included in reports of observational studies

|  | Item No. | Recommendation | Page  No. | Relevant text from manuscript |
| --- | --- | --- | --- | --- |
| **Title and abstract** | 1 | (*a*) Indicate the study’s design with a commonly used term in the title or the abstract | 2 |  |
|  |  | (*b*) Provide in the abstract an informative and balanced summary of what was done and what was found | 2 |  |
| Introduction | | | |  |
| Background/rationale | 2 | Explain the scientific background and rationale for the investigation being reported | 3 |  |
| Objectives | 3 | State specific objectives, including any prespecified hypotheses | 3, 4 |  |
| Methods | | | |  |
| Study design | 4 | Present key elements of study design early in the paper | 4 |  |
| Setting | 5 | Describe the setting, locations, and relevant dates, including periods of recruitment, exposure, follow-up, and data collection | 4 |  |
| Participants | 6 | 1. *Cohort study*—Give the eligibility criteria, and the sources and methods of selection of participants. Describe methods of follow-up   *Case-control study*—Give the eligibility criteria, and the sources and methods of case ascertainment and control selection. Give the rationale for the choice of cases and controls  *Cross-sectional study*—Give the eligibility criteria, and the sources and methods of selection of participants | 4 |  |
|  |  | (*b*) *Cohort study*—For matched studies, give matching criteria and number of exposed and unexposed  *Case-control study*—For matched studies, give matching criteria and the number of controls per case | 4 |  |
| Variables | 7 | Clearly define all outcomes, exposures, predictors, potential confounders, and effect modifiers. Give diagnostic criteria, if applicable | 4, 5 |  |
| Data sources/ measurement | 8* | For each variable of interest, give sources of data and details of methods of assessment (measurement). Describe comparability of assessment methods if there is more than one group | 4, 5 |  |
| Bias | 9 | Describe any efforts to address potential sources of bias | 5, 6 |  |
| Study size | 10 | Explain how the study size was arrived at | 4, 5 |  |
| Quantitative variables | 11 | Explain how quantitative variables were handled in the analyses. If applicable, describe which groupings were chosen and why | 5 |  |
| Statistical methods | 12 | (*a*) Describe all statistical methods, including those used to control for confounding | 5, 6 |  |
|  |  | (*b*) Describe any methods used to examine subgroups and interactions | 5, 6 |  |
|  |  | (*c*) Explain how missing data were addressed | 5, 6 |  |
|  |  | (*d*) *Cohort study*—If applicable, explain how loss to follow-up was addressed  *Case-control study*—If applicable, explain how matching of cases and controls was addressed  *Cross-sectional study*—If applicable, describe analytical methods taking account of sampling strategy | 5, 6 |  |
|  |  | (*e*) Describe any sensitivity analyses | 5, 6 |  |
| Results |  |  |  |  |
| Participants | 13* | (a) Report numbers of individuals at each stage of study—eg numbers potentially eligible, examined for eligibility, confirmed eligible, included in the study, completing follow-up, and analyzed | 6, 7 |  |
|  |  | (b) Give reasons for non-participation at each stage | 6, 7 |  |
|  |  | (c) Consider use of a flow diagram | NA |  |
| Descriptive data | 14* | (a) Give characteristics of study participants (e.g. demographic, clinical, social) and information on exposures and potential confounders | 7 and Table 1 |  |
|  |  | (b) Indicate number of participants with missing data for each variable of interest | Table 1 |  |
|  |  | (c) *Cohort study*—Summarize follow-up time (e.g., average and total amount) | 7, 8 |  |
| Outcome data | 15* | *Cohort study*—Report numbers of outcome events or summary measures over time | 7, 8 |  |
|  |  | *Case-control study—*Report numbers in each exposure category, or summary measures of exposure |  |  |
|  |  | *Cross-sectional study—*Report numbers of outcome events or summary measures |  |  |
| Main results | 16 | (*a*) Give unadjusted estimates and, if applicable, confounder-adjusted estimates and their precision (e.g., 95% confidence interval). Make clear which confounders were adjusted for and why they were included | 7, 8 and Table 2 |  |
|  |  | (*b*) Report category boundaries when continuous variables were categorized | 7, 8 and Table 2 |  |
|  |  | (*c*) If relevant, consider translating estimates of relative risk into absolute risk for a meaningful time period | 8, 9 and Figure 2 |  |
| Other analyses | 17 | Report other analyses done—e.g. analyses of subgroups and interactions, and sensitivity analyses | 8, 9 |  |
| Discussion |  |  |  |  |
| Key results | 18 | Summarize key results with reference to study objectives | 9 |  |
| Limitations | 19 | Discuss limitations of the study, taking into account sources of potential bias or imprecision. Discuss both direction and magnitude of any potential bias | 12, 13 |  |
| Interpretation | 20 | Give a cautious overall interpretation of results considering objectives, limitations, multiplicity of analyses, results from similar studies, and other relevant evidence | 9, 10, 11, 12 |  |
| Generalizability | 21 | Discuss the generalizability (external validity) of the study results | 10, 11, 12 |  |
| Other information |  |  |  |  |
| Funding | 22 | Give the source of funding and the role of the funders for the present study and, if applicable, for the original study on which the present article is based | 13 |  |

**Supplementary Table 2. STROBE checklist.**

|  | Missing values, n (%) | Quartile 1 (≤ 52 years) (n = 273) | Quartile 2 (> 52 – 60 years) (n = 249) | Quartile 3 (> 60 – 69 years) (n = 264) | Quartile 4 (> 69 years) (n = 257) | p value | P_trend_ |
| --- | --- | --- | --- | --- | --- | --- | --- |
| Causes of death, n (%)  Anoxic brain injury  Cardiovascular  MOF  Sepsis  Other | 0 (0.0) | n = 55  11 (20.0)  13 (23.6)  25 (45.5)  3 (5.5)  3 (5.5) | n = 67  6 (9.0)  24 (35.8)  29 (43.3)  3 (4.5)  5 (7.5) | n = 85  5 (5.9)  32 (37.6)  38 (44.7)  0 (0.0)  10 (11.8) | n = 89  6 (6.7)  37 (41.6)  35 (39.3)  4 (4.5)  7 (7.9) | 0.09 | 0.01  < 0.01  0.50  0.55  0.51 |
| Length of mechanical ventilation, days, median (IQR) | 129 (12.4) | 6.0 (2.0 – 14.0) | 7.0 (2.0 – 14.0) | 5.0 (1.0 – 12.0) | 1.0 (0.0 – 5.0) | < 0.01 | < 0.01 |
| Length of stay in intensive care unit, days, median (IQR) | 343 (32.9) | 16.0 (7.0 – 34.0) | 15.0 (7.0 – 29.0) | 9.0 (5.0 – 15.0) | 6.0 (4.0 – 16.0) | < 0.01 | < 0.01 |
| Length of hospitalization, days, median (IQR) | 321 (30.8) | 37.0 (22.0 – 45.0) | 34.0 (23.0 – 40.0) | 26.0 (14.0 – 34.0) | 20.0 (13.0 – 30.0) | < 0.01 | < 0.01 |
| Time to ECMO escalation, days, median (IQR) | 0 (0.0) | 2.0 (1.0 – 4.5) | 1.0 (0.0 – 3.0) | 1.0 (0.0 – 2.0) | 0.5 (0.0 – 1.0) | 0.17 | < 0.01 |
| Hemorrhagic complication, n (%)  ENT  Digestive  Urological  Hematoma  Other | 32 (3.1) | 90 (34.1)  16 (17.8)  23 (25.6)  1 (1.1)  41 (45.6)  9 (10.0) | 87 (36.0)  19 (21.8)  32 (36.8)  3 (3.4)  26 (29.9)  7 (8.0) | 78 (31.0)  11 (14.1)  27 (34.6)  4 (5.1)  25 (32.1)  11 (14.1) | 50 (19.8)  7 (14.0)  7 (14.0)  3 (6.0)  13 (26.0)  20 (40.0) | < 0.01 | < 0.01  0.37  0.36  0.09  0.02  < 0.01 |
| Device-related infection, n (%)  Positive aerobic anaerobic blood culture  Positive documented device analysis  Documented infection at implantation site | 355 (34.0)  355 (34.0)  382 (36.6) | 48 (21.9)  7 (3.2)  8 (3.7) | 33 (17.2)  3 (1.6)  3 (1.6) | 26 (13.8)  6 (3.2)  4 (2.3) | 6 (6.7)  1 (1.1)  1 (1.1) | < 0.01  0.59  0.54 | < 0.01  0.53  0.20 |
| Complications, n (%)  De novo aortic or mitral regurgitation  Tamponade  Ventricular tachycardia or cardiac arrest | 372 (35.7)  5 (0.5)  5 (0.5) | 20 (9.8)  13 (4.8)  31 (11.4) | 26 (13.7)  8 (3.2)  35 (14.2) | 9 (4.9)  14 (5.3)  32 (12.2) | 3 (3.3)  3 (1.2)  39 (15.2) | < 0.01  0.052  0.56 | 0.01  0.09  0.31 |
| Ischemic stroke  Hemorrhagic stroke  Mesenteric ischemia | 353 (33.8)  353 (33.8)  354 (33.9) | 15 (6.8)  12 (5.5)  12 (5.5) | 27 (14.1)  12 (6.3)  10 (5.2) | 21 (11.2)  8 (4.2)  7 (3.7) | 2 (2.2)  3 (3.3)  4 (4.4) | < 0.01  0.72  0.86 | 0.59  0.33  0.47 |

**Supplementary Table 3. In-hospital outcomes according to age quartiles.**

ECMO, extracorporeal membrane oxygenation; ENT, Ear, nose and throat; IQR, interquartile range; MOF, multi-organ failure

|  | Quartile 1 (≤ 52 years) | Quartile 2 (> 52 – 60 years) | Quartile 3 (> 60 – 69 years) | Quartile 4 (> 69 years) | p value | Ptrend |
| --- | --- | --- | --- | --- | --- | --- |
| **30-day mortality** | | | | | | |
| AMICS | 27/123 (22.0) | 41/168 (24.4) | 59/185 (31.9) | 57/158 (36.1) | 0.03 | < 0.01 |
| Electrical storm | 3/19 (15.8) | 2/16 (12.5) | 9/30 (30.0) | 4/10 (40.0) | 0.33 | < 0.01 |
| Post-cardiotomy | 6/19 (31.6) | 4/16 (25.0) | 8/16 (50.0) | 5/15 (33.3) | 0.53 | 0.51 |
| Fulminant myocarditis | 8/35 (22.9) | 1/3 (33.3) | 1/3 (33.3) | 1/1 (100.0) | 0.50 | < 0.01 |
| Acute-on-chronic heart failure | 4/49 (8.2) | 6/28 (21.4) | 5/24 (20.8) | 3/9 (33.3) | 0.11 | < 0.01 |
| Acute valvular heart disease | 1/4 (25.0) | 1/9 (11.1) | 2/3 (66.7) | 1/4 (25.0) | 0.27 | 0.47 |
| **1-year mortality** | | | | | | |
| AMICS | 34/123 (27.6) | 51/168 (30.4) | 81/185 (43.8) | 75/158 (47.5) | < 0.01 | < 0.01 |
| Electrical storm | 3/19 (15.8) | 2/16 (15.2) | 14/30 (46.7) | 7/10 (70.0) | 0.01 | < 0.01 |
| Post-cardiotomy | 7/19 (36.8) | 9/16 (56.2) | 10/16 (62.5) | 7/15 (46.7) | 0.47 | 0.54 |
| Fulminant myocarditis | 8/35 (22.9) | 1/3 (33.3) | 1/3 (33.3) | 1/1 (100.0) | 0.51 | < 0.01 |
| Acute-on-chronic heart failure | 7/49 (14.3) | 7/28 (25.0) | 6/24 (25.0) | 3/9 (33.3) | 0.42 | 0.02 |
| Acute valvular heart disease | 1/4 (25.0) | 1/9 (11.1) | 2/3 (66.7) | 1/4 (25.0) | 0.26 | 0.47 |

**Supplementary Table 4. 30-Day and 1-Year All-Cause Mortality Rates by Shock Etiology and Age Quartile**

Data are presented as number of deaths/total patients in each subgroup (%). The total number of deaths presented in this table may not correspond to the totals reported in Table 2, as a small number of patients had no etiology recorded in the dataset.


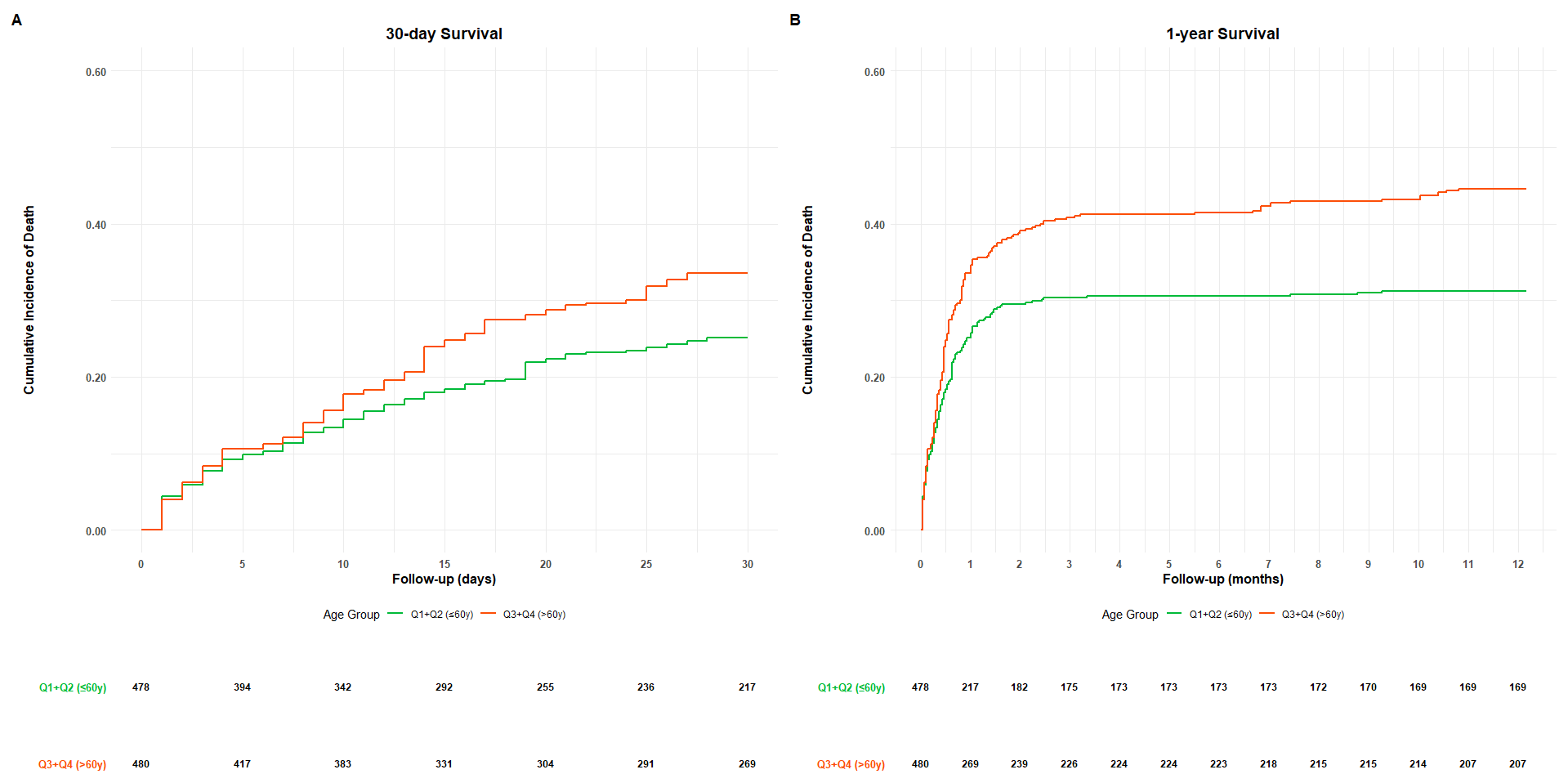


Supplementary Figure 1. Cumulative Incidence of Death According to Binary Age Groups in Patients with Cardiogenic Shock Supported with Microaxial Flow Pump


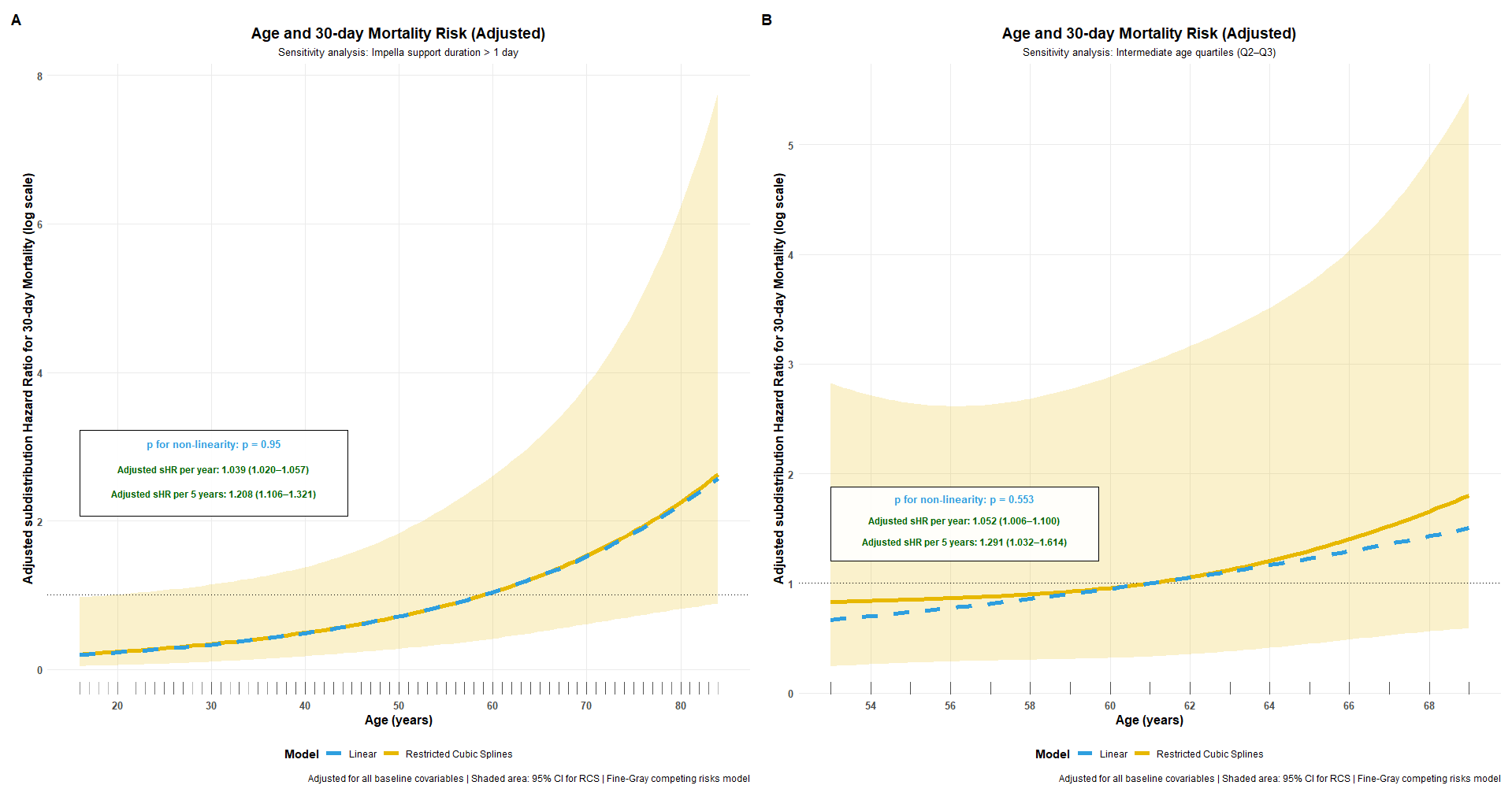


Supplementary Figure 2. Relationship Between Age and Adjusted 30-day Mortality Risk Using Restricted Cubic Splines — Sensitivity Analyses

CI, confidence interval; RCS, restricted cubic spline; sHR, subdistribution hazard ratio
